# TLRs and RAGE are elevated in carotid plaques from patients with moderate-to-severe obstructive sleep apnea syndrome

**DOI:** 10.1007/s11325-020-02029-w

**Published:** 2020-02-20

**Authors:** Wioletta Olejarz, Alicja Głuszko, Agata Cyran, Katarzyna Bednarek-Rajewska, Robert Proczka, David F. Smith, Stacey L. Ishman, Ewa Migacz, Wojciech Kukwa

**Affiliations:** 1 Department of Biochemistry and Pharmacogenomics, Faculty of Pharmacy, Medical University of Warsaw, Warsaw, Poland; 2 Centre for Preclinical Research, Medical University of Warsaw, 02-097 Warsaw, Poland; 3 Chair and Department of Biochemistry, Faculty of Medicine, Medical University of Warsaw, Warsaw, Poland; 4 Department of Pathology, Medical University of Warsaw, Warsaw, Poland; 5 Department of Clinical Pathology, Poznan University of Medical Sciences, Poznan, Poland; 6 Cardiology Center Jozefow, American Heart of Poland, Warsaw, Poland; 7 Divisions of Pediatric Otolaryngology and Pulmonary and Sleep Medicine, Cincinnati Children’s Hospital Medical Center, Cincinnati, OH 45229 USA; 8 Department of Otolaryngology-Head and Neck Surgery, University of Cincinnati School of Medicine, Cincinnati, OH 45229 USA; 9 Department of Otorhinolaryngology, Faculty of Dental Medicine, Medical University of Warsaw, Warsaw, Poland

**Keywords:** Obstructive sleep apnea, RAGE, TLR, Intermittent hypoxia, Atherosclerosis

## Abstract

**Background:**

There is growing evidence that obstructive sleep apnea (OSA) promotes vascular endothelial dysfunction and atherogenesis. Pathways that mediate this pathology may include Toll-like receptors (TLRs) and receptor for advanced glycation end products (RAGE) which play a significant role in proinflammatory processes. The aim of this study was to measure the expression of the above-mentioned receptors in relation to OSA severity in carotid plaques obtained during open endarterectomy.

**Methods:**

This prospective study included patients with a sleep study prior to surgery and a plaque specimen obtained during standard open endarterectomy. Immunohistochemistry of TLR2, TLR4, TLR7, TLR9, RAGE, HMGB1, and NF-κB was performed on atherosclerotic plaques from carotid arteries of patients with and without OSA.

**Results:**

There were 46 patients (22 women, mean age 73.2 ± 1.3 years): 14 control patients, 13 with mild, 11 with moderate, and 8 with severe OSA. The expression of all TLRs and RAGE increased proportionately with increasing OSA severity. The largest differences between patients with severe OSA and no OSA were found for TLR2 (2.88 ± 0.35 vs. 1.27 ± 0.47, *p* < 0.001), TLR4 (2.88 ± 0.35 vs. 1.64 ± 0.5, *p* < 0.001), TLR9 (2.38 ± 0.52 vs. 1.45 ± 0.52, *p* < 0.01), and RAGE (2.5 ± 0.53 vs. 1.82 ± 0.6, *p* < 0.05).

**Conclusion:**

TLR2, TLR4, TLR9, and RAGE expression was significantly increased in carotid plaques of patients with moderate-to-severe OSA when compared with control patients with no OSA and those with mild OSA. TLR and RAGE-mediated pathways may play a significant role in OSA-dependent atherogenesis.

## Introduction

**Fig. 1:**
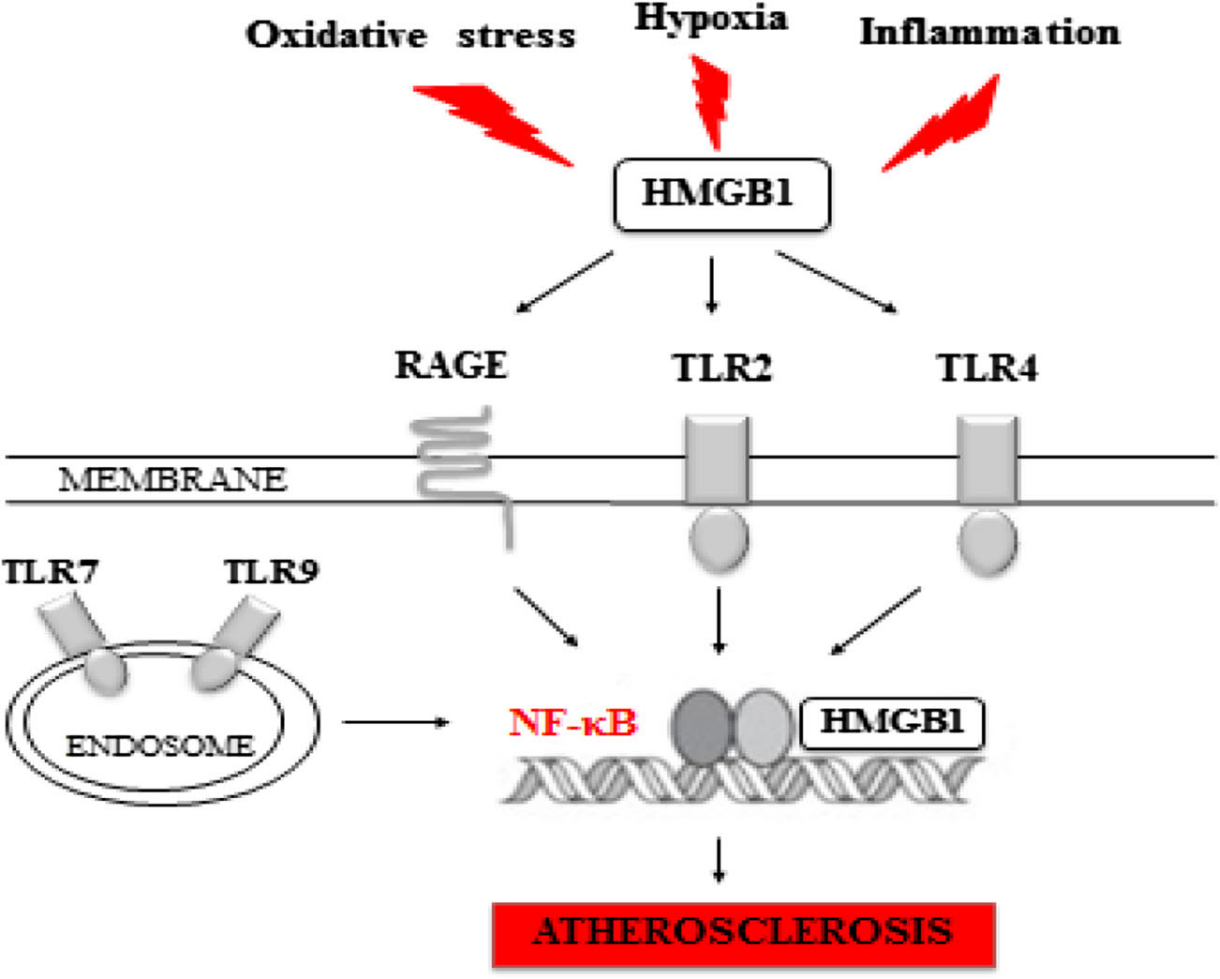
Effect of intermittent hypoxia, oxidative stress, and inflammation on activation of nuclear factor-κB (NF-κB) by stimulation of receptor for advanced glycation end products (RAGE) and Toll-like receptors (TLRs) through extracellular high-mobility group box 1 protein (HMGB1). Nuclear HMGB1 acts as a DNA chaperone with DNA binding and bending activities and regulates replication, recombination, repair, and transcription

Sleep significantly influences cardiovascular regulation. Obstructive sleep apnea (OSA) is a severe form of sleep-disordered breathing (SDB), which is independently associated with the development and progression of cardiovascular disease (CVD), including atherosclerosis. The association between OSA and atherosclerosis has been investigated for years, but the molecular mechanisms contributing to the pathogenesis are still not known [1]. Numerous studies have demonstrated impaired endothelial function and signs of early atherosclerosis, such as increased carotid intima-media thickness, in OSA patients and improvement of those parameters with use of continuous positive airway pressure (CPAP) therapy [2, 3]. Sympathetic activation, systemic inflammation, intermittent hypoxia, and oxidative stress are the main intermediary mechanisms linking OSA and CVD [4]. Inflammation in atherosclerotic plaques attenuates the tensile strength of the collagen cap, enhances cell death and reinforces prothrombotic activity, and causes acute coronary syndrome [5]. Human studies showing the correlation of OSA severity with plaque formation were based on various diagnostic techniques or instrumentation, including ultrasonography [6, 7], cardiovascular magnetic resonance (CMR) [8], and recently PET/MRI [9]. Arterial stiffness has also been used as an indicator of early signs of atherosclerosis [10, 11]. Intermittent hypoxia induces vascular alterations and chronic inflammation which are common pathways for atherosclerosis formation and plaque destabilization [12]. During episodes of chronic inflammation, the stimulation of Toll-like receptors (TLRs) and the receptor for advanced glycation end products (RAGE) may occur. TLRs are expressed by macrophages, neutrophils, and dendritic cells and are intimately tied to the process of atheroma formation [13]. Some TLRs, especially TLR2 and TLR4, are called “atherogenic promoters.” They are activated by high-mobility group box 1 protein (HMGB1) which leads to the activation of the nuclear factor NF-κB [14] (Fig. 1). Interaction of HMGB1 with TLRs and RAGE stimulates signaling pathways such as SAPK/JNK (stress-activated protein kinase/c-Jun NH2-terminal kinase), p38 MAPK (mitogen-activated protein kinase), and ERK1/2 (Ras-extracellular signal-regulated kinase 1/2), consequently leading to NF-휅B activation and overexpression of cytokines, adhesive molecules, and matrix metalloproteinases (MMPs) linked to atherogenesis [15].

With this in mind, our aim was to evaluate if the severity of OSA determines the levels of expression of TLRs and RAGE, as well as HMGB1 and NF-휅B, in human atherosclerotic plaque samples removed during open endarterectomy.

## Materials and methods

### Patients and criteria

This was a prospective study enrolling consecutive patients scheduled for endarterectomy from March 2014 to July 2017. Inclusion criteria included completion of endarterectomy, plaque sampling, and presence of a preoperative sleep study. Exclusion criteria were as follows: BMI > 35 kg/m^2^, smoking > 20 years, diabetes mellitus treatment > 5 years, and previous treatment for OSA. This study was approved by the Bioethical Committee of the Medical University of Warsaw. Written informed consent was obtained from each patient.

### Sleep study

OSA diagnosis was established prior to surgery and was based on a home sleep apnea test (HSAT) using the WatchPAT™ (Itamar Medical) portable sleep apnea diagnostic system. The WatchPAT system measures peripheral arterial tonometry, oximetry, heart rate, actigraphy, body position, and snoring. The severity of OSA was classified by the apnea hypopnea index: mild (defined as an AHI of 5–< 15 events/h), moderate (AHI of 15–< 30 events/h), and severe (AHI ≥ 30 events/h). Those with an AHI < 5 events/h were considered controls. The Epworth sleepiness scale (ESS) was also obtained for all patients. Other recorded clinicopathological parameters included body mass index (BMI) and neck and waist circumferences.

### Atherosclerotic plaque

Atherosclerotic plaques were obtained from patients undergoing open carotid endarterectomy. Patients with non-occlusive high-grade atherosclerotic stenosis measuring > 70% luminal narrowing and a history of ipsilateral stroke or transient ischemic attacks (TIA) were scheduled for endarterectomy. These indications were based on the North American Symptomatic Carotid Endarterectomy Trial (NASCET) and European Carotid Surgery Trial (ECST) guidelines.

### Antibodies

The following antibodies were used in the study: rabbit anti-human TLR2 (ab213676 at dilution 1:200), mouse anti-human TLR4 (ab22048 at dilution 1:100), rabbit anti-human TLR7 (ab124928 at dilution 1:100), mouse anti-human TLR9 (ab134368 at dilution 1:200), rabbit anti-human RAGE (ab3611 at dilution 1:100), rabbit anti-human HMGB1 (ab79823 at dilution 1:400), and rabbit anti-human NF-κB p65 (ab16502 at dilution 1:1000) (all from Abcam, UK).

### Immunohistochemistry

Immunohistochemistry was performed on biopsies of formalin-fixed, paraffin-embedded human atherosclerotic plaques. The sections were subsequently incubated with optimally diluted primary antibody or without primary antibody (negative control) overnight at 4 °C. Tissues were irrigated with wash buffer (PBST), and endogenous peroxidase activity was quenched with a peroxidase suppressor. Detection was performed using a biotin-conjugated secondary antibody (post-primary rabbit and mouse IgG Novocastra REF7111) and streptavidin-horseradish peroxidase (streptavidin-HRP) followed by colorimetric detection using diaminobenzidine (DAB). Sections were incubated with the substrate/chromogen, DAB, prepared from DAB Chromogen (1.74% w/v diaminobenzidine, in a stabilizer solution), and Novolink DAB Substrate Buffer (Polymer). The slides were assessed by the light microscope ZEISS Observer Z1 (Axiovision 4.8 software; illumination system LUMEN 200; PRIOR) at the total magnification × 200. Results were interpreted using a light microscope and aided in the differential diagnosis of pathophysiological processes, which may or may not be associated with a particular antigen.

### Evaluation of immunohistochemistry

Results are expressed as a percentage of positive cells in the field of view (FOV), evaluating five randomly chosen high-power fields (HPF × 200 magnification). The number of positive cells was evaluated according to the following 4-tiered classification: 0, negative reaction (less than 10% of stained cells); 1, low expression (11–50% of cells stained positive); 2, intermediate expression (51–75% of cells stained positive); 3, high expression (over 75% of cells stained positive). Masking of the pathologist to the experimental groups was performed to eliminate bias.

### Statistical analysis

The results are expressed as the mean ± standard deviation (SD). Comparisons were made using ANOVA with Tukey post hoc. Differences between experimental groups were considered statistically significant at *p* < 0.05. Statistical analyses were performed with the SPSS 23.0 software (SPSS).

## Results

### Patient demographics

Sixty-eight patients underwent endarterectomy, and 46 subjects met inclusion criteria; these 46 had a mean age 73.2 ± 8.7 years. The control group (*n* = 14) included those patients with an AHI < 5 events/h. There were 13 patients (28%) with mild OSA, 11 (24%) with moderate OSA, and 8 (18%) with severe OSA (Table 1). Patients with severe OSA had larger neck and waist circumferences (42 ± 2.5 cm and 110.8 ± 6.8 cm, respectively) and BMIs (31.3 ± 3.5 kg/m^2^) compared with those with mild and moderate OSA (38.6 ± 3.9 cm, 97.6 ± 6.5 cm, and 26.8 ± 2.8 kg/m^2^ and 39.5 ± 2.2 cm, 100.3 ± 13.4 cm, and 28.6 ± 3.6 kg/m^2^, respectively) (*p* < 0.05).

**Table 1:** Patient demographic data and home sleep apnea test (HSAT) results in 4 groups of patients divided according to pAHI

	Control	Mild	Moderate	Severe
Cases, *n*	14	13	11	8
Age, years	71.4 ± 8.1	73.8 ± 7.2	74.3 ± 11.2	73.4 ± 8.3
Female/Male, n	9/5	7/6	3/8	3/5
BMI, kg/m^2^	26.0 ± 2.7	26.8 ± 2.8	28.6 ± 3.6	31.3 ± 3.5*
Neck circumference, cm	37.4 ± 3.9	38.6 ± 3.9	39.5 ± 2.2	42 ± 2.5*
Waist circumference, cm	97.7 ± 11.3	97.6 ± 6.5	100.3 ± 13.4	110.8 ± 6.8*
pAHI, events/h	3.6 ± 1.3	9.0 ± 2.2***	21.7 ± 3.1***	43.5 ± 13.3***
ODI	1.3 ± 0.6	3.5 ± 1.7***	13.5 ± 4.5***	33.1 ± 9.3***
ESS score	3.4 ± 1.5	7.5 ± 8.1	7.8 ± 4.8*	8.7 ± 6.2**

*BMI*, body mass index; *pAHI*, peripheral arterial tone apnea/hypopnea index; *ODI*, oxygen desaturation index; *ESS*, Epworth sleepiness scale

^*^p < 0.05, ^**^p < 0.01, ^***^p < 0.001 vs. control

### Inflammatory markers are upregulated in atherosclerotic plaques from patients with OSA

**Fig. 2:**
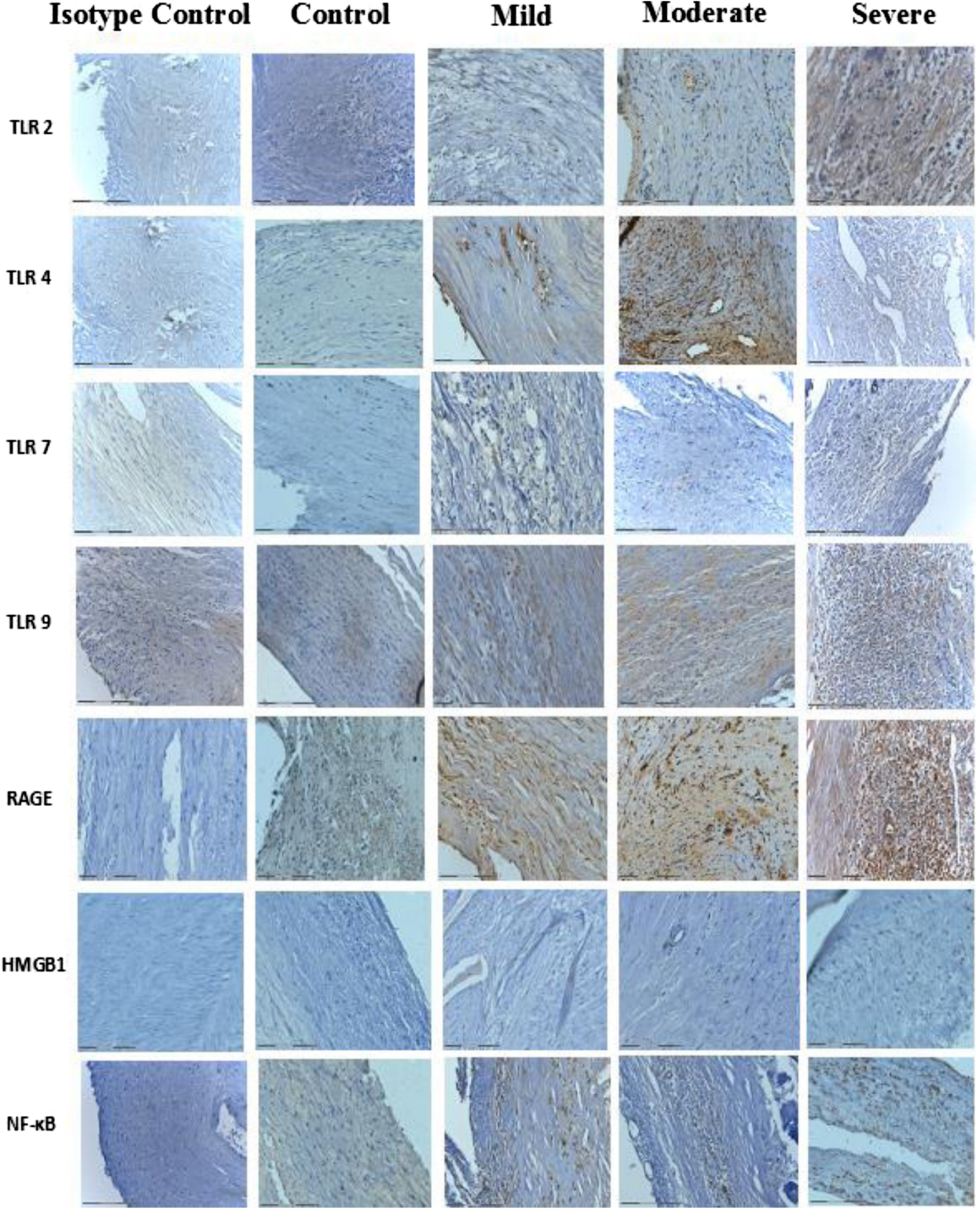
Immunohistochemical analysis of Toll-like receptor (TLR) 2, 4, 7, and 9; receptor for advanced glycation end products (RAGE); high-mobility group box 1 protein (HMGB1); and nuclear factor-κB (NF-κB) in carotid atherosclerotic plaque in patients undergoing carotid endarterectomy. Immunohistochemistry staining was performed with rabbit or mouse antibodies and with isotype control IgG. Original magnification × 200. Scale bar = 200 μm

**Fig. 3:**
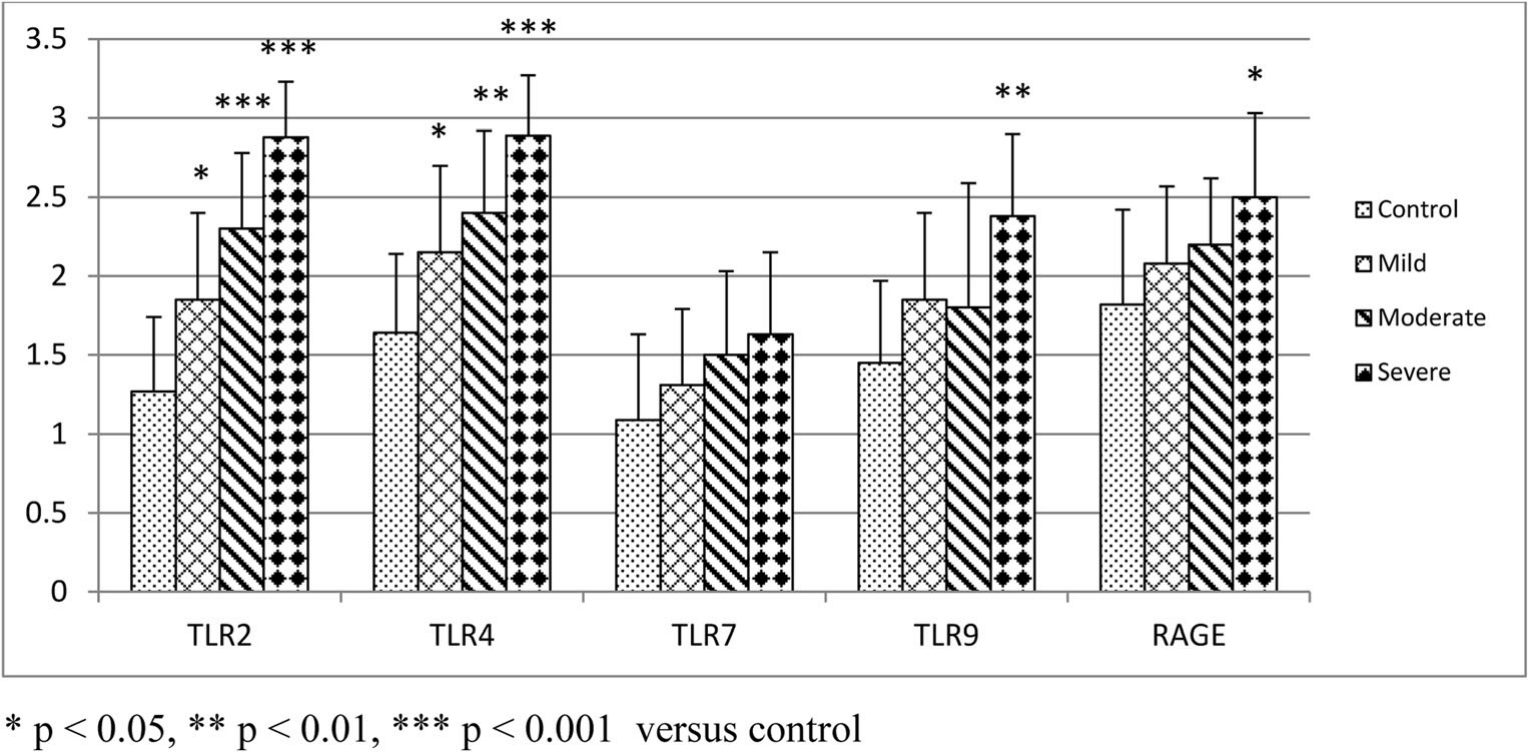
Immunohistochemistry of Toll-like receptors (TLR) and receptor for advanced glycation end products (RAGE)

**Fig. 4:**
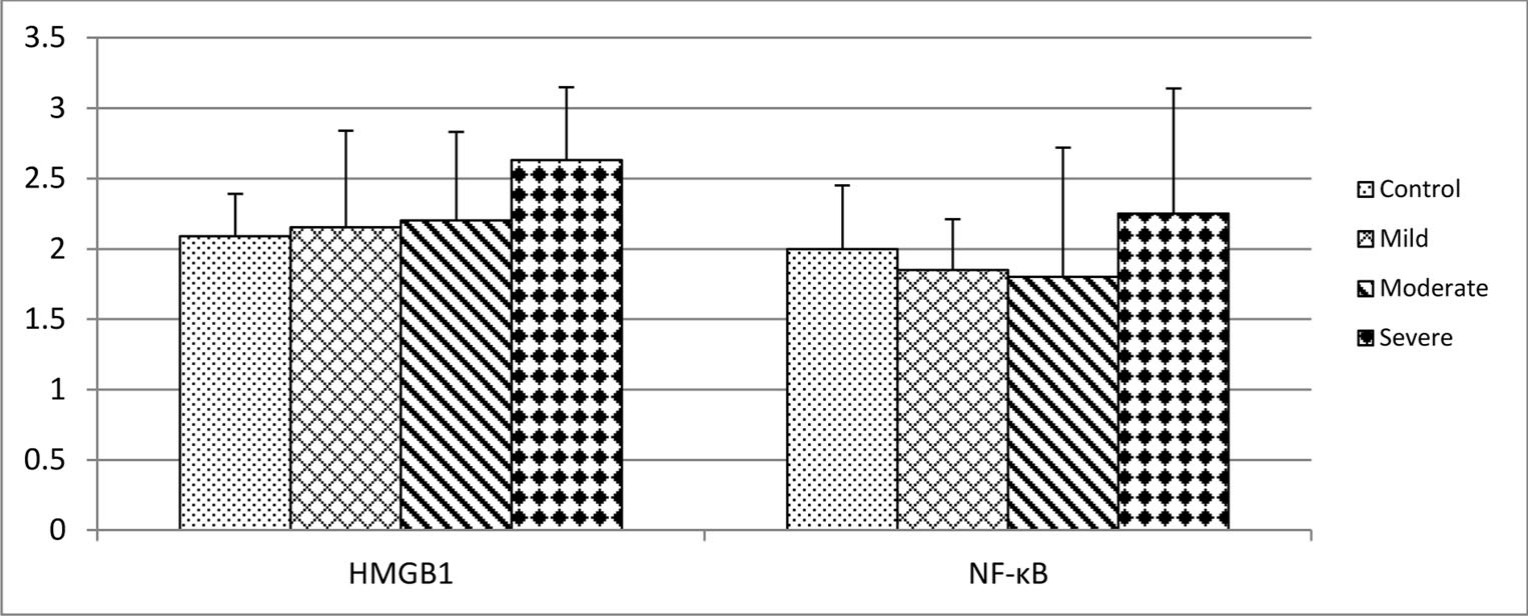
Immunohistochemistry of high-mobility group box 1 protein (HMGB1) and nuclear factor-κB (NF-κB)

Expression of TLR2 and TLR4 was grossly increased in atherosclerotic plaques in mild, moderate, and severe OSA (Fig. 2). TLR2, TLR4, TLR7, TLR9, and RAGE were predominantly expressed in the cytoplasm. In contrast, HMGB1 and NF-κB were observed in the nuclei. Expression of these receptors was increased in endothelial cells and macrophages. Expression of TLR9 and RAGE was significantly increased in atherosclerotic plaques from patients with severe OSA (Fig. 2). In patients with mild OSA, expression of TLR2 and TLR4 was intermediate (*p* < 0.05), while in moderate and severe OSA, expression of TLR2 and TLR4 was high (*p* < 0.001) (Fig. 3). TLR9 and RAGE were significantly increased in patients with severe OSA (*p* < 0.01 and *p* < 0.05, respectively) (Fig. 3). HMGB1 and NF-κB were positive (high expression in > 75% cells) in atherosclerotic plaques in severe OSA (Fig. 4). Immunohistochemistry of all the investigated receptors, HMGB1, and NF-κB in the control group and those with mild, moderate, and severe OSA are presented in Table 2.

**Table 2:** Immunohistochemistry of Toll-like receptors (TLR), receptor for advanced glycation end products (RAGE), high-mobility group box 1 protein (HMGB1), and nuclear factor-κB (NF-κB)

	Control	Mild	Moderate	Severe
TLR2	1.27 ± 0.47	1.85 ± 0.55*	2.3 ± 0.48***	2.88 ± 0.35***
TLR4	1.64 ± 0.5	2.15 ± 0.55*	2.4 ± 0.52**	2.88 ± 0.35***
TLR7	1.09 ± 0.54	1.31 ± 0.48	1.5 ± 0.53	1.63 ± 0.52
TLR9	1.45 ± 0.52	1.85 ± 0.55	1.8 ± 0.79	2.38 ± 0.52**
RAGE	1.82 ± 0.6	2.08 ± 0.49	2.2 ± 0.42	2.5 ± 0.53*
HMGB1	2.09 ± 0.3	2.15 ± 0.69	2.2 ± 0.63	2.63 ± 0.52
NF-κB	2.0 ± 0.45	1.85 ± 0.36	1.8 ± 0.92	2.25 ± 0.89

^*^p < 0.05, ^**^p < 0.01, ^***^p < 0.001 vs. control

## Discussion

In the present study, we demonstrate the potential role of TLRs and RAGE in the development of atherosclerotic lesions in patients with OSA. Intermittent hypoxia in these patients may promote atherosclerosis by stimulating RAGE and TLRs, leading to plaque destabilization. All analyzed receptors were expressed in the cytoplasm of cells that make up atherosclerotic plaques, while the intensity of HMGB1 and NF-κB staining was increased substantially in the nucleus. TLR2 and TLR4 frequently colocalized with NF-κB, a transcription factor that induces TLR expression but also mediates the downstream signaling of TLR on ligand engagement. Similarly, the presence of NF-κB was also observed in TLR-positive cells. These results confirm that the NF-κB pathway is activated in the atherosclerotic plaques. Our research indicates that TLR2, TLR4, TLR9, and RAGE were strongly expressed in patients with severe OSA, which may be associated with carotid plaque destabilization. Therefore, these receptors may become ideal pharmacologic targets for preventing progression of OSA-induced atherosclerosis.

Accumulating evidence indicates that TLRs are associated with the development and progression of atherosclerosis [16]. It was shown that exogenous TLR2 and TLR4 activation increases atherosclerotic plaque formation and the plaque-media ratio [17]. Seimon et al. found TLR2 and CD36-dependent apoptosis and necrotic plaque core in macrophages undergoing endoplasmic reticulum-induced stress [18]. Mullick et al. showed reduction of atherosclerosis in LDLR-deficient (LDLR−/−) mice with a complete deficiency of TLR2 [19]. Higashimori et al. also demonstrated diminished foam cell accumulation in lesion-prone areas of the aorta of ApoE−/− mice with TLR2 deficiency [20]. In lesions of both ApoE-knockout mice and human coronary bypass grafts, endogenous TLR4 ligands are present, implicating a role for TLR expression in atherosclerosis [21]. Katsagyris et al. demonstrated enhanced TLR4 expression in endothelial cells of carotid atheroma in patients with unstable carotid plaques [22]. TLR7 and TLR9 possess a specific ability to induce type I interferon (IFN) production. TLR9 is activated by CpG motifs in nucleic acids that are released during vascular necrosis and has been closely linked to the development of atherosclerotic lesions [23]. Attenuated expression of RAGE ligands (HMGB1, S100B) and decreased expression of matrix metalloproteinases and adhesion molecules in the aorta were demonstrated in Apo E−/− and RAGE−/− double knockout mice [24]. Increased expression of RAGE in aortas was observed in a separate study in Apo E −/− knockout mice [25]. Burke et al. demonstrated that necrotic core expansion, thinning of the fibrous cap, and plaque instability are associated with increased expression of RAGE [26]. Therefore, TLRs and RAGE may provide a link between innate and adaptive immunity and enhance the cellular immune response in the plaque.

HMGB1 is a DNA chaperone released into the extracellular space by apoptotic cells and plays a significant role in inflammation [27]. It is overexpressed and released from atherosclerotic endothelial cells, vascular smooth muscle cells, foam cells, macrophages, and activated platelets [28]. Our results suggest that NF-κB activation may be involved in HMGB1–RAGE-mediated atherosclerosis in OSA. Also, Liu et al. confirmed HMGB1 activity in atherosclerotic plaque formation in a transgenic mouse model of atherosclerosis (apolipoprotein E-deficient mice) through RAGE [29].

Few studies analyzed the role of TLRs in atherogenesis in relation to OSA. Akinnusi et al. found increases in TLR4 expression, NF-κB nuclear binding, and release of IFNγ, TNF-α, and IL-6 in circulating monocytes in OSA [30]. The present study of TLRs and RAGE was largely confined to macrophages. TLRs and RAGE in macrophages permit local differentiation of these cells into antigen-presenting cells (APCs) because receptor ligation stimulates this process. TLR and RAGE activation occurs as a primary event, subsequently leading to NF-κB activation. Our results indicated a nuclear accumulation of NF-κB in atherosclerotic plaque in patients with OSA. NF-κB is considered as one of the significant inflammatory factors in the pathogenesis of atherosclerosis. Israel et al. previously demonstrated increased proinflammatory NF-κB-dependent genes in OSA patients through NF-κB activation [31].

These results confirm the important role of inflammation in the cardiometabolic consequences of OSA. A growing body of evidence indicates that OSA is independently associated with atherogenesis [32, 33].

There are other studies suggesting a relationship between habitual snoring and atherosclerotic plaque formation. Lee et al. was the first to show that heavy snoring could increase the risk of carotid atherosclerosis [34]. Unlike that study, we found no correlation between the expression of analyzed receptors and snoring severity measured as a percentage of sleep (above 45 dB).

As with any study, there are a few limitations worth discussing. First, the sample size is small which could limit our statistical analyses. Second, we did not collect data regarding BMI in 8 patients which limited our ability to study the correlation of the receptors’ expression and BMI. Lastly, we did not perform morphological sizing of the plaques by ultrasound. As we only collected plaque samples, the structure of the arterial wall could not be analyzed. Future studies should analyze the relationship of the plaque and the vessel wall both in ultrasound and in immunohistochemical analyses. This will allow us to determine if increased expression is also associated with increased plaque formation.

## Conclusions

The severity of OSA is associated with significant increases in TLR2, TLR4, TLR9, and RAGE in human carotid plaque. This, in turn, may activate the increased proinflammatory NF-κB-dependent genes in OSA patients. As many studies have shown that these receptors play a pivotal role in carotid plaque instability, our findings suggest that targeting these pathways could provide future therapeutic options for OSA patients.
